# Nearing Dental Anesthesia

**Published:** 1912-11-15

**Authors:** 


### NEARING DENTAL ANESTHESIA.

"Painless Dentistry" is a phrase which probably was invented when dental operations were first attempted, and it has lived to this day as the embodiment in words of a great human need on the one hand and, upon the other, as a kind of jocular Will-o-the-wisp, not to be overtaken by man. In late years, however, encouraging progress has been made toward practicable dental anesthesia. Cataphoresis, though not immediately successful, opened a wide path for investigators; then followed "pressure anesthesia," the admixture of nitrous-oxid and oxygen, and local injections of novocain and adrenalin. The "dental obtundents" put upon the market from time to time, many of them partially useful, have been innumerable. It is an open question as to whether the dental profession deserves reproach for having moved so tardily toward this important end. The production of localized anesthesia by the penetration of dense tooth structure—in the control of, without injury to, the vital elements of the dentin—is an achievement far more difficult of compass than that of general anesthesia. It is a blessing, indeed, that relief came first and most easily for the entire body; our lesser and more intricate problem has been delayed by greater natural difficulties. But reproach will justly and speedily come in the future to operators who fail to use the means now available, and daily becoming more simple of application.

The administration of ether or chloroform for dental operations is prohibitive because of the danger, and the necessary preparation and care of the patient during each administration. The usual programme of ether anesthesia carried out through the filling of a dozen simple cavities would be a criminal absurdity. The nitrous-oxid-oxygen method has come to our help as comparatively safe and simple in those cases of extreme nervous excitability where more than localized anesthesia is needed. Jameson[1] has reported recently very gratifying results from this method.

From present appearances, our ultimate success in obliterating pain during routine dental operations will lie in the simplified use of the nitrous-oxid-oxygen method, or by the control of the dental pulp by local injections which penetrate the cancellous structure of the alveolus and thence enter the circulation at the apical foramina. In all probability both methods will be considered desirable to meet varying conditions.

The work of Fischer[1], Ottesen[2] and others has resulted in a comparatively safe, sure and convenient technique of local anesthesia by the injection of novocain and adrenalin or suprarenin.

Three methods chiefly are used: (1) The needle is carried through the gum tissue and against the cancellous alveolar process; (2) a sensory nerve trunk is reached or approximated by the needle, passing through one of the namy foramina; (3) "intra-alveolar" injection—an opening, the caliber of the needle, having been drilled through the outer plate of the alveolar process, the needle is carried deeply into that structure and near the apex of the root of the tooth to be operated upon. Dr. Ottesen's article, already noted, describes very clearly the steps to be followed.

Too great care cannot be taken in the maintenance of asepsis during every step of the use of the syringe. This is self-evident, of course; but just as the niceties of cavity preparation for inlay work have led to higher standards of care, so the necessity for a sterile field and *sterile* instruments in this work will have a salutary influence among even our best operators. The difficulties of attaining oral asepsis during routine operations have laid dentists open to a rather sweeping indictment of negligence from physicians and from the public. This charge often is inconsiderate from a lack of knowledge on the critic's part of the mechanical and other obstacles to be overcome, and of the curious and blessed

---

1Guido Fischer, Local Anesthesia in Dentistry, Translated by R. H. Riethmuller. Lea & Febiger, Philadelphia, 1912.

2T. I. Ottesen, Local Anesthesia in Dental Surgery. Sept. 1912 number of The Journal of Allied Societies.

fact of comparative "mouth immunity," which shields the patient from perpetual infection in that ever-septic cavity. How many millions of open alveoli, unprotected after extraction of the teeth, heal beautifully though filled with putrescent food particles and luxuriant bacteria? What percentage of that number of such wounds, averaging three-quarters of an inch in depth, three-eighths in width, crammed with filth, in any part of the body, would heal promptly and fill with healthy tissue? But because of this most fortunate circumstance we must not infer that the operator is excused from any care within his reach further to protect the patient from infection. The danger of oral sepsis is always present. The desirability of the best possible surgical technique is undeniable, and the imparative demand for such methods in the operations which are being considered should lead to improvements along this line in general practice.

It seems not unreasonable to believe that dental anesthesia is wholly feasible. That it will not be needed, nor advisable, in the majority of dental operations is probable; but in the future there should be little excuse for real suffering, or for poor cavity preparation—which so long has been the alternative.—Editorial in *Journal of Allied Societies,* Sept. 1912.

